# Maud L. Menten: Pioneering Physician and Biochemist

**DOI:** 10.7759/cureus.68054

**Published:** 2024-08-28

**Authors:** Aarnav Sadaria, Pooja Kanyadan, Chitra Kanyadan

**Affiliations:** 1 Biology, Wheeler High School, Marietta, USA; 2 Clinical and Translational Sciences, University of Rochester, Rochester, USA; 3 Internal Medicine, Wellstar Health System, Marietta, USA

**Keywords:** therapeutics, pharmacology, phd, scarlet fever, women in anesthesiology, enzyme kinetics, pediatric pathology, biochemistry

## Abstract

Dr. Maud Leanora Menten, an esteemed Canadian physician, biochemist, and organic chemist, conducted a wide range of valuable biochemistry research for over 40 years, making groundbreaking discoveries about cancer treatments, enzyme kinematics, anesthesia medicine, bacterial toxins, vitamin deficiencies, hematology, and histochemistry. Menten demonstrated intense perseverance and tenacity in her education, defying societal norms to not only become one of the first Canadian women to earn a research-intensive Doctor of Medicine (MD) degree, but to also be one of the first to earn a PhD. Although she was restricted in her work in Canada, she moved to the U.S. and published an estimated 100 research studies over her career. She is most well known for her work with Dr. Leonor Michaelis, with whom she created the Michaelis-Menten equation for the relationship between reaction rate and enzyme-substrate concentration. However, she conducted many other noteworthy research projects, such as using radium bromide for cancer treatment in rats and using electrophoretic mobility to study human hemoglobin, which allowed for a more advanced protein analysis. Her research in hemoglobin preceded the findings of Linus Pauling by several years, however, he is often the only one credited for this work. After her death, the extent and depth of her work was better understood and appreciated by many, and she was recognized by her alma mater, the University of Toronto, and her former workplace, the University of Pittsburgh. She was also posthumously inducted into the Canadian Medical Hall of Fame.

## Introduction and background

**Figure 1:**
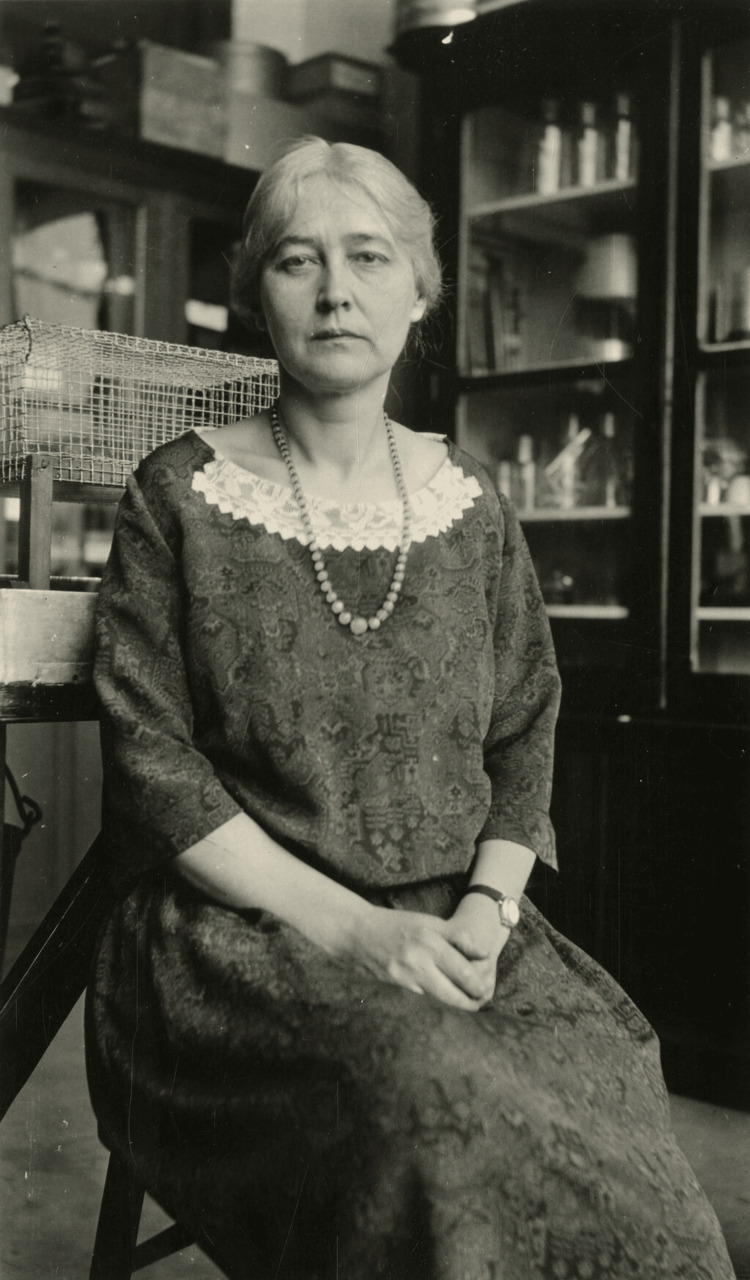
A seated portrait of Dr. Maud L. Menten This image is available through the Smithsonian Institution Archives and on Wikimedia Commons. This image is part of the public domain and has no copyright restrictions, per the Smithsonian Institution [8].

In a time when women were prevented from attaining higher degrees and careers, Dr. Maud Leonora Menten (Figure 1) broke these boundaries by becoming not only one of the first female physicians, but also earning a PhD in biochemistry, conducting extensive research, and being a role model for future generations. Her education started in a small, one-room school, but her passion for higher education motivated her to obtain a Master's degree in physiology from the University of Toronto in 1907 [1]. Menten’s love for research brought her to New York’s Rockefeller Institute, where she published the institute’s first-ever monograph detailing the results of her research on the use of radium bromide for cancer treatment in rats, which marked the start of her impressive achievements [2]. She then returned to the University of Toronto and graduated with a doctor of medicine degree in 1911, which made her one of the first female physicians in Canada [3].

Afterwards, Menten worked with Dr. Leonor Michaelis, one of the world’s leading biochemists at the time. Together, they created the Michaelis-Menten equation, which represents the relationship between reaction rate and enzyme-substrate concentration. An enzyme is a protein that binds to substrate molecule(s) and helps speed up reactions involving those molecules. Enzymes are critical for all bodily functions, thus, the Michaelis-Menten equation is one of the most important equations in all of biochemistry [4]. Menten then went on to become one of the first women to graduate with a PhD in biochemistry from the University of Chicago [2]. Her first job as a pathologist was at the Elizabeth Steele Magee Hospital in Pittsburgh, following which she was promoted from being a demonstrator in pathology to eventually becoming a professor at the University of Pittsburgh School of Medicine [5-6]. She was also hired as a pathologist at the Children’s Hospital of Pittsburgh and served as their laboratory director until her retirement [5-7] while continuing her research career in biochemistry. By the end of Menten’s career, she had published over 100 research studies and articles in biochemistry and pediatrics, motivating generations of women to pursue their dreams regardless of how many obstacles they may face [2]. Dr. Menten’s groundbreaking work will forever be remembered in the scientific community.

## Review

Early life and career

Dr. Menten was a scientific genius. Along with teaching and working as a pediatric pathologist, she spent her lifetime researching across many fields in medicine and biochemistry. Her research lay the groundwork for many important fields, including biochemistry, pathology, and drug therapy.

Maud was born on March 20, 1879, in Port Lambton Ontario, to her father William Menten and mother Elizabeth Menten [4]. Menten spent her childhood in a remote region of Canada. When Menten was 10 years old her family moved to Harrison Mills, British Columbia [2] in the prospects of job opportunities due to the westward expansion of the Canadian Pacific Railroad. After moving to Harrison Mills, Menten and her younger brother were homeschooled by her mother. Three years after their move, a new one-room school opened in Chilliwack, British Columbia. Menten and her brother took a ferry and traveled two-and-a-half miles each way to study here [7]. Despite the daily challenges of getting to school, Menten’s parents' early encouragement of her education laid the foundation of her love for learning and brilliant career.

Menten attended the University of Toronto, where she completed a BA in natural science and English with honors in 1904. While studying for her BA, she worked for two years as an assistant demonstrator with her physiology professor Archibald Macallum. Together, they published research discussing the distribution of chlorides in nerve cells and fibers in 1906 [3, 9]. While working in Dr. Macallum’s lab, Menten also independently studied and wrote a paper on the distribution of fat, chlorides, phosphates, potassium, and iron in striated muscle [3, 10]. In 1906, Menten was inducted into the university’s Sigma Chapter of the Kappa Alpha Theta sorority [7]. She graduated with a Master's degree in physiology in 1907 [1]. Unfortunately, Menten was unable to find employment in the field of research as a woman in Canada. She refused to let this deter her and joined New York’s Rockefeller Institute to conduct medical research [3]. Along with the pathologists Simon Flexner and James W. Jobling, she published the institute’s first-ever monograph, titled “Tumors of Animals'', which detailed the results of their research on the use of radium bromide for cancer treatment in rats in 1910 [2, 7]. After this, she returned to the University of Toronto to receive her medical degree in 1911, becoming one of the first Canadian women to earn a doctorate degree [1]. 

Despite becoming one of the first female Canadian physicians, Dr. Menten had a lack of opportunities due to her gender. Nonetheless, she was motivated to study further, research, and publish her work by all means possible. Thus, her career was a remarkable journey across different institutions and continents. She moved to Western Reserve University in Cleveland and worked in the laboratory of Dr. George Crile, the founder of Cleveland Clinic [1, 7]. Here, Dr. Menten studied hydrogen-ion concentration in the blood to control acid-base balance under anesthesia influence [1, 7]. After graduating from medical school, she traveled at her own expense to Berlin in 1912 to work with one of the world’s leading biochemists, Dr. Leonor Michaelis, at the University of Berlin [2]. Together, they created the Michaelis-Menten equation, which represents the relationship between reaction rate and enzyme-substrate concentration and is one of the most important equations in the field of biochemistry [4].

From 1914 and 1916, Menten worked at Western Reserve University, where she researched alkalinity on malignancy and other pathological conditions at the Barnard Free Skin and Cancer Hospital in St. Louis, Missouri. The findings were published in the *Journal of Cancer Research *[11]. She also obtained her PhD in biochemistry from the University of Chicago in 1916 [12]. During her PhD, she studied the effects of adrenaline on blood and published her findings in the *American Journal of Physiology* [13]. After earning her PhD, Menten began working as a pathologist at the Elizabeth Steele Magee Hospital, a charitable women’s hospital in Pittsburgh, where she stayed from 1916 to 1918 [7]. During this time, Menten researched the pathophysiology and biochemical changes occurring in pathological conditions. She published at least one paper during this time, titled “Positive Wassermann Reaction which Changes to a Negative at Termination of Pregnancy” [7, 14]. In 1918, she was hired as a demonstrator in pathology at the University of Pittsburgh School of Medicine in 1918 [7], and was promoted to assistant professor in 1923 and associate professor in 1926 [5-6]. That same year, she was hired as a pathologist at the Children’s Hospital of Pittsburgh and served as their laboratory director until 1950 [5-7], while continuing her research career in biochemistry. During this time, she rose to the rank of professor just before her retirement. Dr. Maud Menten continued her prolific career both as a clinician-researcher, pathologist, and teacher at Pittsburg. Throughout her career at the University of Pittsburgh, she collaborated with several researchers and produced noteworthy results [15]. From discovering the effects of *Salmonella* endotoxins on blood sugar levels, Menten then went on to discover the subclinical effects of vitamin C deficiency on guinea pigs exposed to diphtheria toxin [7, 15]. She was able to observe that deficiency of vitamin C in these animals led to pathological lesions, including hyperplastic arteriosclerosis and hyperglycemia associated with degeneration of pancreatic islets [3, 7, 15]. She also worked on the prevention of scarlet fever, during which she and her colleagues continued to try new antibiotics to treat children’s pneumonia in 1939 [16]. 

Menten not only contributed to society through research but also inspired further generations of scientists [17]. She spoke at the Women’s Medical Society of Pittsburgh meetings and at the Sigma Sigma Epsilon women’s fraternity at Pitt and served as the fraternity’s faculty advisor [7]. Raising money for her fellow women, Menten was actively involved in the American Association of University Women’s multi-year drive to raise money for 25 fellowships, each amounting to $40,000 [7].

The Michaelis-Menten equation

One of Menten's most famous works is the research she conducted with Dr. Leonor Michaelis on enzyme kinetics. In 1912, Menten left the laboratory of Dr. George Crile to work with biochemist Dr. Leonor Michaelis in Berlin [18]. She was a research assistant in his lab [18]. Building on the enzyme reaction initially proposed by Dr. Victor Henri, Michaelis and Menten developed an equation within the year of 1912 [3, 18]. They were studying the decomposition of sucrose into fructose and glucose via the enzyme invertase with the hope of determining a relationship between reaction rate and enzyme-substrate concentration, for which Menten was largely responsible for measuring the concentration of sucrose and velocity measurements so that they could determine the inversion reaction time [18]. Their equation describes the velocity of enzymatic reactions and represents the relationship between reaction rate and enzyme-substrate concentration [3-4, 18]. According to the hypothesis for the equation, the velocity of the enzymatic reaction on the Y-axis and the concentration of the substrate on the X-axis are directly related, so that the reaction curve has a hyperbolic shape [4]. The derivation of this reaction curve produces the Michaelis-Menten equation:

$V=(VM [S]) / (KM + [S])$

In this equation, V represents the reaction velocity, S represents the substrate concentration, VM represents the maximal reaction velocity, and KM represents the Michaelis constant [4].

In addition to her major contributions to this research, Menten learned German in order to work with Michaelis, as the paper was originally written and published in German [18]. 

The development of the Michaelis-Menten equation had broad impacts that are still important today. Before this, the understanding of enzyme kinetics was not very well defined. This equation allowed for specific and precise measurements of how substrate concentration affects enzyme activity and the rate of reaction [4, 7]. It explains how an enzyme can enhance the kinetic rate of a reaction [3-4]. In addition, prior to the development of this equation, drug developments were not systematic and after this equation, identifying which enzymes needed to be targeted in the development of drugs became more systematic [4, 18-19]. Furthermore, their contribution led to understanding enzyme regulation, studying fast reactions, understanding mechanisms for enzyme catalysis, understanding the properties of single molecules, and drug development [18]. The Michaelis-Menten equation is valuable in drug development as it helps determine how drugs are metabolized in the body [19]. By using the equation, researchers can predict the behavior of drugs that are enzyme inducers such as phenytoin, and which drugs are enzyme inhibitors such as metronidazole. The reaction order changing from mixed to zero or first order can help determine dosage amounts for the drug and the frequency at which those dosages are taken [18-19].

Although there is a limitation to this equation - that is, it does not apply when a substrate is inhibited or activated as a result of a second substrate molecule being bound - several researchers have built on this limitation and furthered our understanding of enzyme-substrate biochemistry [18-19].

Scarlet fever epidemic

Several years after revolutionizing the field of biochemistry with the Michaelis- Menten equation, Menten began to work at Children’s Hospital after being promoted to a pathologist in 1926 [7]. At this time, and especially during the 1930s and 1940s, scarlet fever was a virulent disease with a death rate of 44.7 per 100,000 [7]. In 1925, Menten researched the effects of salmonella on rabbits alongside Dr. Helen M. Manning. They observed that rabbits infected with salmonella became hyperglycemic, which was because of a specific endotoxin shortening the hepatic glycogen stores. Applying this in the 1930s, Menten began her research on developing a vaccine for the scarlet fever toxin [7]. At the same time, researchers at the University of Chicago found that scarlet fever was caused by streptococci bacteria found in the throats of patients [7]. Menten discovered a specific toxin in the streptococci bacteria that could be targeted in scarlet fever patients [7]. Menten then began purifying this toxin for over five years to the point where the substance was 10,000 times more concentrated than any purified toxin previously [7]. It could be determined whether or not children are susceptible to scarlet fever using the Dick test, developed by George and Gladys Dick, which can also be used after immunization with the *Streptococcus* toxin to determine how susceptible the children remain [7, 20]. This immunization proved to be highly effective; by 1941, the death rate plummeted to 0.045 deaths out of 100,000 individuals [7, 20]. During the epidemic, Menten wrote an article in the Pittsburgh Post-Gazette about how the Dick test worked, so that it could be understood by all readers regardless of their educational background [20]. This demonstrates that she was not only a brilliant researcher, but she also cared for and made a significant impact on the daily lives of common citizens by saving many of them from scarlet fever and ensuring they understood the importance of testing and immunization. Menten, along with her colleagues, published her research in scarlet fever immunization in the *Journal of Immunology* in 1939 [20].

Other achievements and discoveries

Throughout her career, Menten faced challenges due to her gender. She was forced to immigrate due to a lack of opportunities in Canada, and the jobs she held were below her ability and degrees. She was promoted to the status of a full professor only near retirement. Despite these challenges, Dr. Menten’s true passion lay in medicine and research. She continued to research even after retirement. She was an inspiring teacher who inspired medical students, residents, physicians, and research associates to give their best efforts in their work [3, 17].

Menten continuously sought out opportunities to learn and research. This is evident in her wide-ranging, cutting-edge research. Although this makes it difficult to individually review and discuss all of her research papers, a list of her key research projects, collaborators, and their impacts has been compiled in Table 1 below.

**Table 1:** List of Maud Menten's most notable research projects and their impacts This table outlines the most noteworthy research projects conducted by Dr. Menten, along with her collaborators and the impacts of the projects. This list was adapted from various sources [1, 5, 7, 9, 15, 17-20].

Year	Research/Discovery	Collaborators	Significance
1907	Studied the effect of radium bromide on cancer tumors in rats [7].	MJ Bancroft	Provided insight into the use of radium bromide in cancer treatment. This paved the way for further cancer research and oncologists [7].
1912	Worked on controlling acid-base balance during anesthesia to avoid surgical shock [7].	George Crile	Improved the conditions of surgery as well as improved patient safety by reducing the risk of surgical shock [7].
1912	Studied the distribution of potassium and chloride ions in nerve cells [7, 9].	Archibald Macallum	Contributed to how electrolytes are distributed throughout the nerve cells [7, 9].
1912	Studied the relationship between reaction rate and enzyme-substrate concentration represented by the Michaelis-Menten equation [17-19].	Leonor Michaelis	Laid the basic groundwork for the subject known as biochemistry and enzyme studies. Also resulted in the ability to develop modern-day drugs [18-19].
1922	Studied the effects of small doses of mercuric chloride on kidneys [7].	DJ Wilson	Found that exposure to mercuric chloride caused kidney lesions [7].
1925	Studied the hyperglycemic effects of *Salmonella* toxins in rabbits [1, 7, 15].	Helen M. Manning	Allowed for the understanding of the effects of *Salmonella* toxins, which helped in refining her technique with purifying toxins and later assisted Menten in developing immunization for scarlet fever [7, 15].
1926	Studied the effects of asphyxia on blood sugar levels in fish [7].	DR Lucas	This study was conducted when fish were important in the study and discovery of insulin, which was one of many projects she conducted while working as a pediatric pathologist [7].
1935	Studied vitamin C deficiency’s effects on bodily response to low levels of diphtheria toxins in guinea pigs [7, 15].	Charles Glen King, Helen M. Manning	Demonstrated the importance of having adequate vitamin C levels, as even before scurvy occurs, low vitamin C levels can be detrimental when the body is affected by toxins [7, 15].
1935 - 1945	Characterized bacterial toxins from *B. paratyphosus*, *Streptococcus scarlatina*, and *Salmonella* to identify what toxin caused scarlet fever [17, 20].	Harold H. Finlay, Aaron H. Stock	These results were used in a successful immunization program against scarlet fever in Pittsburgh in the 1930s - 1940s, which resulted in extremely low death rates. This was a big discovery in early immunization strategies [17, 20].
1943 - 1946	Invented the azo-dye coupling reaction, which allows for histochemical detection of alkaline phosphatase [1, 7, 15].	Josephine Junge, Mary L. Green	Allowed for enzyme activity to be seen for the first time, aiding the further development of disease and enzyme studies [7, 15].
1944	Conducted the first electrophoretic separation of blood hemoglobin proteins, which anticipated the findings of Linus Pauling by several years [5, 7].	Marie A. Andersch, Donald A. Wilson	Pioneered the method for separating proteins, electrophoresis, and furthered protein study, specifically on blood hemoglobin [5, 7].
1951	Studied histochemical distribution of glycogen in the kidneys [7, 15].	Helen M. Manning	This allowed for the understanding of kidney metabolic processes, which is crucial for understanding kidney function [7, 15].

Late career

Menten’s incredible work was only fully recognized by the University of Pittsburgh right before her retirement, and as such, she was promoted to the status of a full professor in 1949 [2]. She retired in 1950 [1]. After her retirement, she pursued oncology research at the British Columbia Medical Research Institute from 1951 to 1954, where she used normal and leukemic mice as animal models to study the nucleic acid content of bone marrow of leukemia patients [2, 7]. By the end of her career, Menten had published over 100 research papers and articles [2]. As arthritis slowly incapacitated her, Menten returned to her hometown of Port Lambton, Ontario, Canada. She passed away on July 17, 1960, in Leamington, Ontario, and was buried in her family plot in Chilliwack, British Columbia [1-4, 7]. Dr. Menten was posthumously inducted into the Canadian Medical Hall of Fame in 1998 [2-4]. She was also given a plaque and statue at her alma mater, the University of Toronto, and was honored at the University of Pittsburgh with memorial lectures and a chair named after her [3, 17].

## Conclusions

Dr. Maud Menten, an astounding physician, biochemist, organic chemist, and researcher, rose above all challenges as a woman living in the early twentieth century to become one of the most achieved and inspirational women for generations to come. Her work was diverse and impactful, demonstrating her love for learning and her dedication to the fields of biochemistry, pathology, and pediatrics. Although it is difficult to uncover and understand all of the work that Dr. Menten completed during her career, as she was often not cited as a co-author or has some work that is only available in print, she was posthumously recognized by the universities that she worked at and her own alma mater, University of Toronto, as one of the most influential physicians in history.
